# COVID-19 second wave and clinical characteristics of cases in Uganda: A retrospective cross-sectional survey of confirmed SARS-CoV-2 cases, March–June 2021

**DOI:** 10.1017/S0950268823001164

**Published:** 2023-07-25

**Authors:** Abel Wilson Walekhwa, Brenda Nakazibwe, Mary Nantongo, Solomon Tsebeni Wafula, Douglas Bulafu, Brenda Ayugi, Caroline Nankabirwa, Godfrey Nsereko, Martha Dorcas Nalweyiso, Tonny Tindyebwa, Roy William Mayega, Abel Bulamu Ekiri, Danstan Bagenda, Monica Musenero, Lawrence Mugisha

**Affiliations:** 1 Science, Technology and Innovation Secretariat- Office of the President, Kampala, Uganda; 2 Makerere University School of Public Health, Kampala, Uganda; 3 Makerere University College of Veterinary Medicine, Animal Resources and Biosecurity, Kampala, Uganda; 4 Ministry of Health, Kampala, Uganda; 5 School of Veterinary Medicine, University of Surrey, UK; 6 University of Nebraska Medical Center, Omaha, NE, USA; 7 Ecohealth Research Group, Conservation and Ecosystem Health Alliance (CEHA), Kampala, Uganda

**Keywords:** asymptomatic patients, COVID-19, characteristics, second wave, Uganda

## Abstract

We conducted a retrospective cross-sectional population-based survey among recovered COVID-19 cases in Uganda to establish the case presentations of the second wave SARS-CoV-2 infections. We interviewed 1,120 recovered COVID-19 cases from 10 selected districts in Uganda. We further conducted 38 key informant interviews with members of the COVID-19 District Taskforce and 19 in-depth interviews among COVID-19 survivors from March to June 2021. Among them, 62% were aged 39 years and below and 51.5% were female with 90.9% under home-based care management. Cases were more prevalent among businesspeople (25.9%), students (16.2%), farmers (16.1%), and health workers (12.4%). Being asymptomatic was found to be associated with not seeking healthcare (APR 2, P < 0.001). The mortality rate was 3.6% mostly among the elderly (6.3%) and 31.3% aged 40 years and above had comorbidities of high blood pressure, diabetes, and asthma. Being asymptomatic, or under home-based care management (HBCM), working/operating/studying at schools, and not being vaccinated were among the major drivers of the second wave of the resurgence of COVID19 in Uganda. Managing future COVID-19 waves calls for proactive efforts for improving homebased care services, ensuring strict observation of SOPs in schools, and increasing the uptake of COVID-19 vaccination.

## Introduction

Coronavirus disease (COVID-19) has remained a Public Health Emergency of International Concern (PHEIC) almost one and half years after the pandemic was declared a PHEIC in January 2020 by the World Health Organization [1]. To date, nations are still under increased pressure to overcome the spiralling global spread of the deadly novel COVID-19, which was responsible for more than 266 million infected individuals and over 5.2 million deaths worldwide as of 7 December 2021 [2, 3]. The wide and unprecedented spread of COVID-19 caused by Severe Acute Respiratory Syndrome Coronavirus 2 (SARS-CoV-2) has been attributed to its ability to spread via respiratory droplets, aerosol, and secretions facilitated by high levels of globalisation and international travel [4].

On 21 March 2020, the Ugandan Ministry of Health reported the first case of confirmed COVID-19 in Uganda from a returning passenger through Entebbe International Airport. Uganda continued to register a few cases of COVID-19 composed mainly of cross-border truck drivers from neighbouring countries until June 2020 when community transmissions increased marking the first wave of COVID-19 cases [5, 6]. To curtail the spread of the disease, the Government of Uganda instituted public health interventions including border closure, institutional lockdown, quarantine, and testing of returnees, contact tracing, and abolishing of public gatherings [7–9]. Following the end of the first COVID-19 wave, which subsided in January–February 2021, most of the instituted control measures were eased, especially lockdown measures such as public transport operations, while others such as worship places and school re-openings were relaxed, allowing the public to resume normal routines that supported their social and economic activities [10, 11]. However, few SARS-CoV-2 infections were still being reported in the communities, and later, unknown factors triggered an exponential rise of COVID-19 cases in different parts of the country, marking the start of the second wave. Most of the cases were reported in the capital city, Kampala, regional cities, and border districts, with over 900 cases daily and reaching a positivity rate of 17% by June 2021 [12]. The period between March and June 2021 is believed to have marked a clear emergence of the second wave of COVID-19 in Uganda with the highest recorded number of cumulative cases of up to 90,000 and with over 2,000 deaths as of 20 July 2021 [5].

Uganda like other countries globally was affected by different variants throughout the period of the COVID-19 outbreak [1].

The variants of concern included B.1.1.7, B.1.351, B.1.617.2, and B.1.525, which had been reported in Nigeria and the UK [2]. These were first observed in Uganda on 5 March 2021 in Kampala and were consistently observed until December 2021 [3]. The A.23.1 was a lineage that emerged in Uganda in the summer of 2020 and later spread in Uganda and globally (Bugembe et al. [13]). The first wave in Uganda was mostly spread by the A.23.1 variant, which later decreased in frequency around February 2021 [3]; later, there was the Omicron variant, which emerged from Wuhan in China [4, 14]. The second wave was mostly aided by B.1.1.7, B.1.351, and B.1.617.2, which had their origins in South Africa and the UK [5]. The second was mostly spread by the Delta variant, which contributed to high hospitalisation since March 2021, and this formed the focus of our investigation. The number of COVID-19 cases in the second wave was strikingly high and more fatal, and there was a high incidence in several districts, but there was no/limited data to explain the factors associated with the observed high incidence of and impact of COVID-19 among the population. To address this gap, we conducted a retrospective cross-sectional study on recovered, confirmed COVID-19 positive PCR-RT/RDT cases from March to June 2021 from 10 districts that had registered the highest number of COVID-19 cases in the second wave to explain the factors associated with the observed spread of COVID-19 among the population [5, 15].

## Materials and methods

### Study Location

The study was conducted in 10 selected districts in Uganda. Uganda is a landlocked country that lies between 1^0^ 29’ South and 4^0^ 12’ North latitude, 29^0^ 34′ East and 35^0^ 0′ East longitude [16]. Uganda has a population of 41.6 million people, based on the Uganda National Household Survey (UNHS) conducted in 2019/20 by the Uganda Bureau of Statistics (UBOS). More than half (54%) of the population is below 18 years of age. Uganda, just like other Sub-Saharan African countries, has a weak healthcare system, characterised by low clinician-to-patient ratio, limited laboratory capacity, poor administration, and limited resources [17, 18].

### Study setting

**Figure 1.**
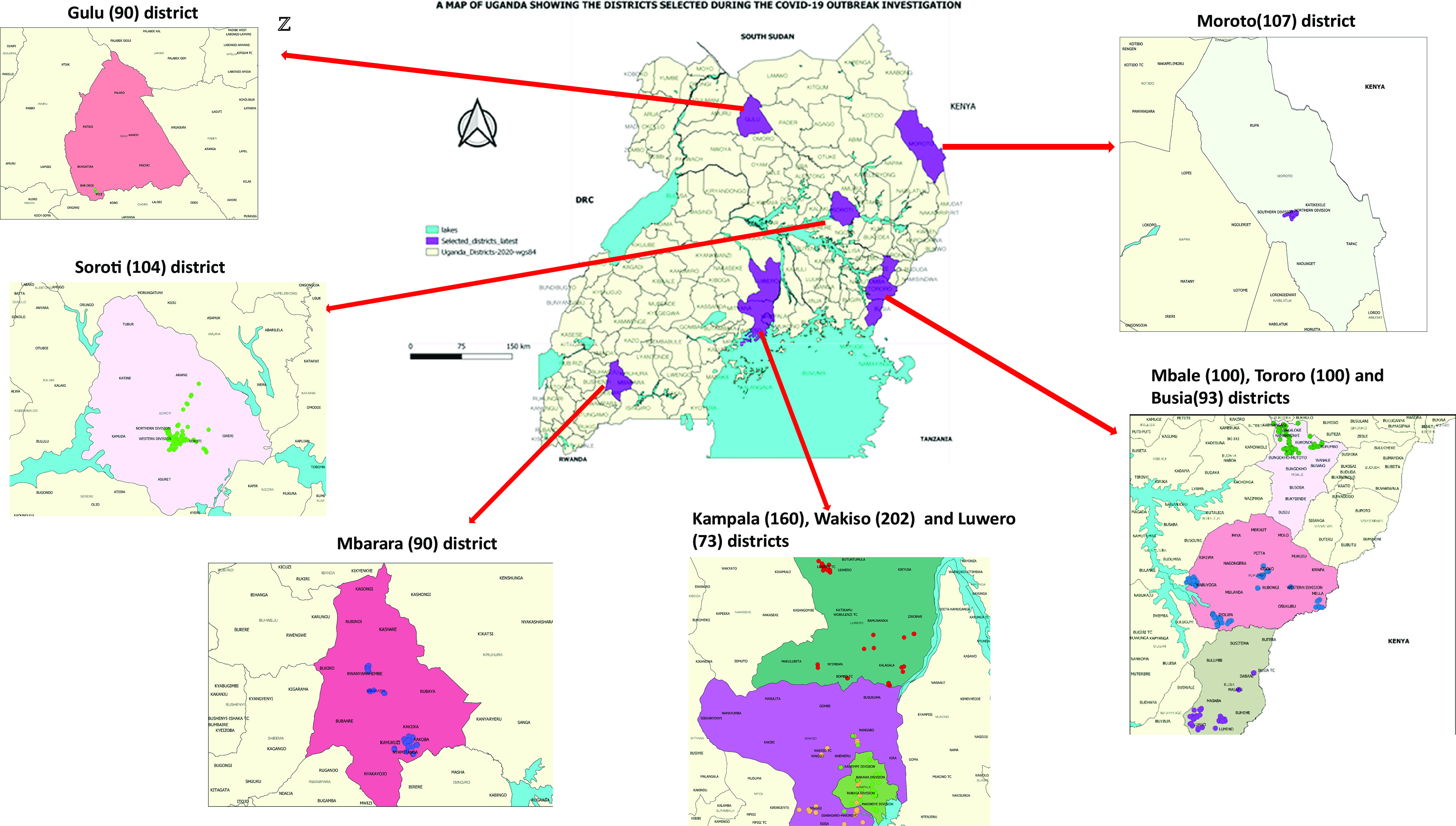
Location of study districts in Uganda.

In this study, we selected 10 districts (Figure 1) representing the main geographic regions that had the highest number of COVID-19 cases as reported by the MOH [5]. The selected districts were the border districts (Busia and Tororo) with Points of Entry (PoE); major road highways for transit of cargo across districts (Mbale, Gulu, Luwero, Soroti, and Moroto districts); and highly populated regional city districts (Wakiso, Gulu, Mbarara, and Kampala) [19, 20].

#### Study population

The study population included patients or caregivers (especially for children below 18 years) of people who had suffered and recovered from COVID-19, either after HBC or after discharge from health facilities. During the investigations, we noted that some of these had died, while for those who were unwell and on treatment, whether at home or in hospitals and on treatment, we interviewed the caretakers/caregivers. The retrospective cross-sectional study was done as part of the outbreak investigation from March 2021 to June 2021 for PCR/RDT confirmed cases.

#### Sample size and sampling procedure

##### Sampling

We selected 10 districts based on their high population densities, high incidences of COVID-19 cases from March 2021 to June 2021 exceeding 300 cumulative cases in the study period, and having PoEs within the districts. We used a computer-based simple random sampling technique [21] to identify 120 COVID-19 cases from each district. This was sampled from the Ministry of Health database of all confirmed and reported COVID-19 cases. On obtaining the sample size, we followed each of the sampled cases, and we used their laboratory investigation forms that were available at respective health facilities in the study districts. We only considered cases that had COVID-19-positive RDT/PCR results (sample form Appendix A). The 1,120 positive COVID-19 PCR tests were done through the routine Ministry of Health testing in a bid to detect COVID-19 among populations. Such people either presented with signs and symptoms related to COVID-19 (suspects) or were contacts of the COVID-19 cases. The Government of Uganda, through the Ministry of Health, made the testing available and mandatory for those who presented as above [6]. This was aimed at identifying cases early and linking them to care in a bid to minimise mortalities. Such people could voluntarily identify themselves to any testing centres for COVID-19 or the community health workers (CHWs) would identify them and refer them for this service. This testing was highly mobilised by the Government of Uganda and the Ministry of Health [7]. The COVID-19 champion was the President of the Republic of Uganda, His Excellency Yoweri Kaguta Museveni, who made several presidential addresses and provided strong political leadership in the bid to fight the COVID-19 pandemic in Uganda [6].

As this was voluntarily done, women sought more care than males according to our results.

The information extracted was then used to systematically sample (Table 1) and locate the recovered COVID-19 cases who were interviewed in the community.

**Table 1.** Recruitment protocol

Confirmed COVID-19 cases (RDT/PCR) in the MOH dashboard on 15^th^ June 2021	67,215
Sample size estimated at national level from 10 high transmission districts	1,200
Eligible populations in high transmission districts	1,200
Accessed population and those who consented to the study	1,120 (93.3%)

We further conducted 38 key informant interviews (KIIs) and 19 in-depth interviews of purposively selected participants in all districts (Appendix C). The key informants comprised COVID-19 District Task Force (DTF) members based on their knowledge and active participation in the COVID-19 outbreak response interventions (Appendix B). In-depth Interviewees were participants who had either had COVID-19 19 and recovered or had lost a COVID-19 case in the family.

### Data collection, management and analysis

#### Data collection and management

##### a) Quantitative data

Quantitative data was collected by trained and experienced epidemiologists using open-ended semi- structured questionnaires (Appendix A uploaded on the mWater portal (@mWater,2021), using an open-source cloud-based web application that was deployed on Android tablets. First, we obtained the details of the case listings in Microsoft Excel from the national database of COVID-19 cases at MOH that guided the selection of target districts with cases ranging from 100–150 cases per district. We then proceeded to the targeted districts to further access records for COVID-19 cases for verification and selection of participants.

The field teams further accessed laboratory investigation forms of the COVID-19 PCR and RDT-positive cases from the laboratories of the selected health facilities in each of the selected districts to extract data on variables such as personal details of the patients such as name, phone number, village, next of kin, and clinical symptoms. The collected information was then used to locate the recovered COVID-19 cases in respective communities guided by the Village Health Teams (VHTs). The selected cases were called via telephone to arrange appointments before the visits. On the day of the visit, the investigation team members took the potential respondents through the consenting process using Appendix D. Data from each participant was collected from a community COVID-19 case interviewer-administered questionnaire that was adopted from the MOH standard tool which assessed the socio-demographics and clinical characteristics of the COVID-19-positive cases. The live COVID-19 cases who consented to the study provided the information, but for those who had died, the next of kin provided this information. The next of kin were also taken through the same consenting process. All the data collected on tablets was uploaded daily onto an mWater portal server secured with passcodes that was only accessed by the principal investigators.

##### b) Qualitative data

An in-depth and key informant guide (Appendix C) was used to conduct interviews with members of the communities in the selected districts who had contracted COVID-19 and the DTF members, respectively. The main theme explored was drivers of the COVID-19 transmissions and spread during the second wave. Verbal consent was obtained from all participants before the commencement of any interview. From each district, four respondents (two male and two female) who had contracted COVID-19 were interviewed during in-depth interviews. Both the KIIs and in-depth interviews were audio-recorded using smartphones and tablets and the audios transcribed verbatim into Microsoft Word, that were only accessed by the study team.

### Data analysis

Quantitative data was exported and cleaned using MS Excel 2016 (Microsoft Corporation, Redmond, WA) and all the data records that had missing data were eliminated at the cleaning stage. Data was analysed using STATA 15.0 statistical software (StataCorp, Texas). Descriptive analyses were performed for demographic characteristics, and clinical characteristics of the COVID-19 cases were presented as frequencies, proportions, and means where appropriate. The outcome variable was binary: being symptomatic (coded 0) or asymptomatic/not symptomatic (coded 1). To assess the association between the outcome variable and the explanatory variables, we considered a generalised linear model of the Poisson family with a logarithm as the conical link function with a robust error variance. This resulted in Crude Prevalence Ratios (CPR) at 95% confidence intervals. Furthermore, variables with a threshold *P*-value less than 0.05 (*P*-value <0.05) at bivariate analyses were subjected to the multivariable regression analyses to adjust for confounding, thus establishing Adjusted Prevalence Ratios (APR). At multivariable analysis, only variables with a *P*-value less than 0.05 were considered significant. Both the CPR and APR have been reported. Qualitative data was analysed using manual thematic analysis, diverging, converging, and emerging themes with representative quotes that were obtained during the analysis. The outputs of these findings are presented in Table 8 in the appendices.

## Results

### Response

A total of 1,120/1,200 RT-PC and RDT COVID-19 confirmed cases from 10 districts in Uganda completed the survey with a 93% response rate.

### Characteristics of COVID 19 cases

#### Social demographics

**Figure 2.**
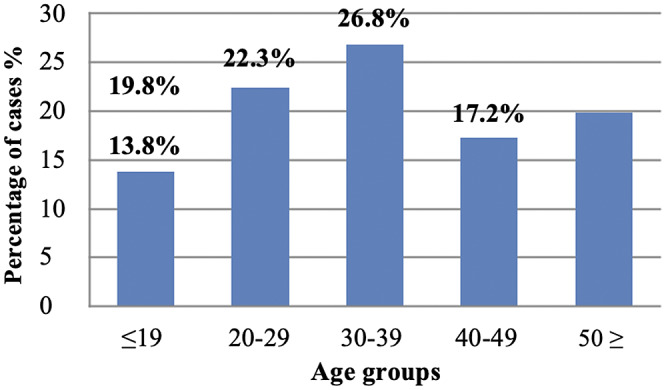
Adjusted age distribution of study participants.

Of the COVID-19 cases interviewed, more than half 51.5% (577/1120) were females. Although we found increased numbers of cases across all age groups, more occurrences were among the young and middle age groups (30–39 years) at 26.8% (300/1120). Overall, we found increased cases of up to 62% among the age group 39 years and below (Figure 2). When we adjusted for age, the majority of the cases were between 40 years and above. We further found that most of the respondents were in the business class, 25.9% (290/1,120), followed by students, 17.2% (193/1,120), farmers/peasants, 17.1% (192/1,120), and health workers, 12.4% (139/1,120). The detailed socio-demographic characteristics of the interviewed cases are summarised in frequencies and percentages (Table 2).

**Table 2.** Socio-demographic characteristics and history of COVID-19 cases

(1) Socio-demographics		
*Variable*	Frequency (*N* = 1,120)	Percentage (%)
*Sex*		
Female	577	51.5
Male	543	48.5
*Age group*		
≤19	155	13.8
20–29	250	22.3
30–39	300	26.8
40–49	193	17.2
50≥	222	19.8
*Number of household members*		
≤5	610	54.5
6–8	345	30.8
(2) History of COVID-19 cases		
*Patient had COVID-19 symptoms at a certain point of illness*		
Yes	895	79.9
No	199	17.8
No response	26	2.32
*Location where symptoms began*		
Where patient sought care **multiple response**		
Hospital	607	65.5
Pharmacy/clinic	159	17.2
Health center	136	14.7
VHT	12	1.3
Church	4	0.4
Traditional healer	1	0.1
Others	35	3.8
Other household members had COVID -like symptoms		
Yes	525	46.9
No	566	50.5
Don’t know	1	0.09
2 weeks before symptom onset or testing, had visited **multiple response**		
Market	188	20.3
Clinics/hospitals	163	17.6
Other mass gathering	127	13.7
Church/mosque	99	10.7
Other towns/districts	99	10.7
Salons	90	9.7
Of those that had been in contact; Setting of exposure to		
contact (*n* = 226)		
Home	104	46.0
Workplace	57	25.2
School	24	10.6
Co-worker	56	25.2
Classmates	20	9.0
Clients	15	6.8
Teacher	4	1.8
Healthcare attendant	2	0.9
Church mate	2	0.9

*Note*: The significance of asterisks are 0.005.

Most respondents had visited various places or attended social gatherings: markets (20.3%), clinics/hospitals (17.6%), places of worship (10.7%), high-risk towns or districts (10.7%), and mass gatherings such as funerals (13.7%) before developing/testing for COVID-19. 21.5% (241/1120) had contacts with COVID-19-like symptomatic persons, while 18.2% (204/1,120) did not have any contact, and 59.9% (671/1,120) did not know of any contact with anyone with COVID- 19-like symptoms 2 weeks before the onset of symptoms (Table 1).

#### Symptoms

**Figure 3.**
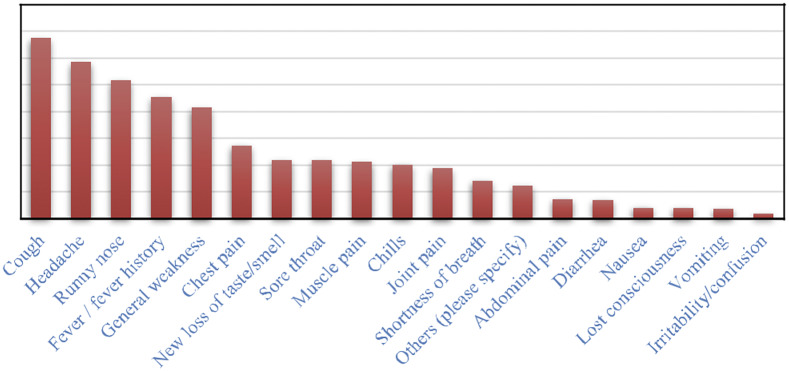
Symptoms experienced during illness.

Most cases (79.9%, 895/1120) acknowledged having developed COVID-19 symptoms at a certain point during the course of illness, while a small proportion (17.8%,199/1120) were asymptomatic (Figure 3).

##### a) Admission status

Only 9.1% of the COVID-19-positive cases were admitted to health facilities (Table 3). According to age group, most cases 31.3% (130) with underlying conditions were aged 40 years and above. However, an increased number of young people (13–39 years), ranging from 13% (12) to 21% (63), reported having underlying conditions (Table 3). Among the cases aged 40 years and above, 31.3% (130) had underlying conditions, and many of them who were admitted either required oxygen, ventilation, or admission to the ICU as summarised in Table 3. The most commonly encountered underlying conditions were high blood pressure, diabetes, and asthma.

**Table 3.** Number of cases, underlying conditions, and need for admission by age group

Age group	Cases	Underlying conditions	Admitted to health facility	Admitted to ICU	Needed oxygen	Needed ventilation	Dead
≤5	16	1(6.2)	0	0	0	0	0
6–12	46	1(2.2)	1(2.2)	0	0	1(16.7)	0
13–19	93	12(13)	2(2.1)	0	0	2(10.5)	1 (1.1)
20–29	250	29(11.6)	11(4.4)	1(1.6)	3(4.8)	4(6.4)	3 (1.2)
30–39	300	63(21)	28(9.3)	7(7.1)	11(11)	4(4.1)	11 (3.7)
40≥	415	130(31.3)	60(14.5)	19(14)	35(25.7)	15(11.3)	26(6.3)

*Note*: Underlying conditions included high blood pressure, diabetes, and asthma.

##### b) Vaccination status

The majority of the cases investigated (78.4% or 878 cases) had not received any COVID-19 vaccine, with only 14.8% (166) having received one dose of AstraZeneca vaccine and only 4.1% (46) with two doses received among the vaccinated group (Table 4). Furthermore, slightly above average (58.7%) participants (542/924) were willing to take the COVID-19 vaccine. The elderly survivors aged 40–49 years (PR = 1.43, 95% CI 1.10–1.84) and ≥ 50 years (PR = 1.52, 95% CI 1.18–1.96) were more willing to receive COVID-19 vaccination.

**Table 4.** Vaccination status of COVID-19 patients

Vaccination status of the cases before testing (*n* = 1,090)	Frequency	Percentage (%)
Not vaccinated at all	878	78.4
One dose of AstraZeneca	166	14.8
Two doses of AstraZeneca	46	4.1
*Reasons for not being vaccinated against COVID-19*		
Concerns about safety of the vaccine	231	39.4
Vaccines are not accessible	226	38.5
Personal reluctancy (still waiting to see others)	97	16.5
Lacked time or transport	33	5.6
*Intention to receive COVID-19 vaccine soon (n = 924)*		
No	382	41.3
Yes	542	58.7

##### c) Survival status

From the data, 3.7% (41/1079) of the cases died of COVID-19 during the second wave. The elderly, 50 years and above, were eight times more likely to die after adjusting the prevalence ratio 8.0 (1.04–61.52). We further noted cases of death in the age group starting from 20–49 years of age but with slightly more numbers among persons who were 30–39 years, represented by the prevalence ratio of 3.8 (CI: 0.47–31.1). Additionally, participants who were vaccinated with at least one dose of the vaccine were six times more likely to survive compared to those not vaccinated as per adjusted prevalence ratio 6.1 (3.24–11.57) as shown in Table 5.

**Table 5.** Factors associated with survival among the COVID-19 cases

		Died (*n* = 41)		Survived (1,079)			
Factor	Variables	No.	%	No	%	Crude PR/95% CI	Adjusted PR/95% CI
Gender							
	M	20	3.7^a^	523	96.3	1.0	
	F	21	3.6^a^	556	96.4	1.0	(0.54–1.80)
Age group							
	≤19	01	0.6	154	99.4	1.0	
	20–29	03	1.2	247	98.8	1.9	(0.20–17.74)
	30–39	11	3.7	289	96.3	5.7	(0.74–43.66)
	40–49	05	2.6	188	97.4	4.0	(0.47–34.05)
	50≥	21	9.5	201	90.5	14.7 (1.99–107.96)	8.0 (1.04–61.52)^b^
Sought care							
	No	04	5.1	74	94.9	1.0	
	Yes	33	3.6	882	96.4	0.7(0.256–1.93)	
Was symptomatic							
	No	04	2.0	195	98.0	1.0	
	Yes	35	3.9	860	96.1	2.0	(0.699–5.41)
Admission status							
	No	22	2.2	996	97.8		
	Yes	19	18.6	83	81.4	8.6	(4.82–15.37)
Vaccination status							
	No	33	3.8	845	96.2	1.0	
	Yes	22	0.4	208	98.1	0.5 (0.18–1.4)	0.5 (0.19–1.39)

a The significance was 0.05 at 95% CI.

b The significance of asterisks are 0.005.

##### d) Being asymptomatic

A small proportion (17.8%, 199/1120) were asymptomatic (Table 1). At bivariate analysis, results showed that not seeking care (CPR 1.99, *P*-value 0.003), not being admitted (CPR 2.15, *P* 0.013), and other household members not having symptoms (CPR 1.52, *P* 0.001) were positively associated with being asymptomatic among the COVID-19 cases. While a household size of greater than nine members (CPR 0.63, *P* 0.025), and having contact with others 2 weeks before testing (CPR 0.38, *P* 0.000) were likely to be symptomatic among the COVID-19 cases. The details of the bivariate analysis are presented in Table 6.

**Table 6.** Showing characteristics of asymptomatic patients

Variable	Outcome		CPR 95% CI	*P*-value	APR 95% CI	*P*-value
	Asymptomatic (*n* = 225)	Symptomatic (*n* = 895)				
Sex						
Male	114 (21)	429	(79)	–		
Female	111 (19.2)	466	(80.8)	0.92(0.725-	0.464	
Age						
≤40	160 (21.7)	578	(78.3)	–		
41≥	65	(17)	317	(83)	0.78 (0.605–1.018)	0.068
≤10	6 (15.4)	33 (84.6)	–			
11–20	38	(26.2)	107	(73.8)	1.70 (0.777–3.736)	
21–30	52	(20.5)	202	(79.5)	1.33 (0.613–2.889)	
31–40	64	(21.3)	236	(78.7)	1.39 (0.643–2.386)	
41≥	65	(17)	317	(83)	1.11(0.513–2.386)	
Household size (members)						
≤5	140 (23)	470(77)	–	–		
6–8	61 (17.7)	284(82.3)	0.77(0.588–1.01)	0.059	0.79 (0.536–1.160)	0.227
9 ≥	24 (14.6)	141(85.4)	0.63 (0.426–0.943)	**0.025**	0.69(0.379–1.276)	0.241
Vaccination status						
No	165 (18.8)	713 (81.2)	–			–
Yes	44 (20.8)	168 (79.2)	1.104 (0.821–1.486)	0.512		
Sought care						
Yes	100 (10.9)	815(89.1)	–	–		
No	17 (21.8)	61 (78.2)	1.99 (1.260 3.157)	**0.003**	2.1 (1.330–3.318)	**0.001**
Admission status						
Yes	10 (9.8)	92 (90.2)			–	
No	214 (21)	803(79)	2.15(1.177–3.914)	**0.013**	3.54 (1.151–10.9)	0.027
Other household members got symptoms						
Yes	78 (14.9)	447(85.1)	–	–	–	
No	128 (22.6)	438(77.4)	1.52(1.179–1.965)	**0.001**	0.72(0.502–1.024)	0.068
Had contact with COVID-19-like symptoms 2 weeks before						
No	64 (31.4)	140(68.6)	–	–	–	
Yes	29 (12)	212(88)	0.38(0.258–0.571)	**0.000**	0.16(0.083–0.309)	**0.000**
Don’t remember	131 (19.5)	540 (80.5)	0.62(0.482–0.803)	**0.000**	0.35(0.244–0.492)	**0.000**

##### e) Health-seeking behaviours

Most respondents (81.7% or 915 cases) sought care after noticing symptoms of COVID-19. A total of 79.4% (823) tested after feeling COVID-19-like signs and symptoms followed by those who had been in contact with a confirmed case (19% or 197 cases). Participants from the central region (prevalence ratio 0.94; 0.94–0.99 95% CI) were less likely to seek care for COVID-19 symptoms, while health workers (PR 1.06; 1.01–1.12) and persons with underlying health problems (PR 1.04; 1.01–1.09) had more proactive health-seeking behaviours (Table 7). Being asymptomatic was found to be associated with not seeking healthcare (APR 2, *P* < 0.001) (Table 6).

**Table 7.** Intention to receive COVID-19 vaccines among health-seeking participants

Variables	Sought care for symptoms adjusted PR (95% CI)	*P*-value	Intention to receive COVID-19 vaccine adjusted PR (95% CI)	*P*-value
Region				
North	1		1	
East	0.98(0.94–1.03)	0.499	1.08 (0.91–1.28)	0.386
Central	**0.94 (0.94–0.99)**	**0.017**	1.08(0.91–1.27)	0.376
West	0.99(0.93–1.05)	0.717	0.99 (0.78–1.26)	0.935
Gender				
Female	1		1	
Male	0.97(0.93–1.00)	0.069	1.02 (0.92–1.14)	0.690
Age group				
≤19	1		1	
20–29	0.98 (0.91–1.05)	0.564	1.17 (0.95–1.43)	0.145
30–39	1.01 (0.93–1.10)	0.676	1.22 (0.95–1.57)	0.115
40–49	1.02 (0.93–1.10)	0.730	**1.43 (1.10–1.84)**	**0.006**
50≥	1.01 (0.93–1.10)	0.826	**1.52 (1.18–1.96)**	**0.001**
Household size				
≤5	1		1	
6–8	1.00 (0.95–1.04)	0.856	0.96 (0.85–1.09)	0.553
9≥	1.03 (0.93–1.07)	0.926	1.07 (0.91–1.25)	0.405
Occupation				
Business	1		1	
Student	1.02 (0.95–1.11)	0.543	**1.33 (1.07–1.67)**	**0.011**
Farmer/peasant	0.99 (0.93–1.05)	0.659	0.86 (0.71–1.05)	0.147
Health worker	**1.06 (1.01–1.12)**	**0.035**	**1.37 (1.16–1.63)**	**<0.001**
Other	1.00 (0.94–1.05)	0.885	1.11 (0.95–1.29)	0.190
Occupations				
Had an underlying health problem				
No	1		1	
Yes	**1.04 (1.01–1.09)**	**0.022**	1.08 (0.94–1.23)	0.289

### Key emerging issues from key informant interviews

Key issues emerged from KIIs that could have contributed to the wide spread of SARS-CoV-2, including

infections under HBCM, social gatherings, myths, misconceptions and misinformation, politics,

schools, weak health systems, and stigma as summarised in Table 8.

**Table 8.** Emerging issues and respondent quotes from KIIs and in-depth COVID-19 interviews

Categories of KII and in-depth interview responses	Summary quotes from respondents
Home-Based Care (HBC) ‘Inadequate surveillance of confirmed cases’ and ‘High costs of facility-based care’ Most of the confirmed cases (90.9%) (1,017/1120) were treated from their homes under HBC. COVID-19 cases under HBC management were reported to have continued to move freely within communities as reported per statement quotes of key informants and in-depth interviews. Some respondents considered hospital care as too expensive while others had mild symptoms, even some were asymptomatic.	*‘…you see when home-based care was introduced, the situation got out of hand because after testing positive for COVID-19, people went back to their workplaces such as the salon or shop even though they had mild symptoms like; flu, fever or cough. So, I think that is the biggest driver….’* Assistant District Health Officer, District A. *‘I was going to be put on oxygen but it was a lot of money, know how long I was going to be there, whether a week. And so, the health worker said that, ‘my advice to you is if you can get some money to buy this medicine, you treat yourself from home.’ So, I went back home. I am here with my wife who also contracted COVID-19 and she got treatment and recovered.’* IDI Male, District W. *‘Since I wasn’t that severely sick, I decided to stay home under home based care.’* IDI 1, District A. *‘…the good thing I was asymptomatic so I isolated from home.*’ IDI 2, District A.
Social Gatherings: ‘Unregulated market vendor movements’, ‘Illegally operating bars’ and ‘Nonadherence to burial COVID-19 SOPs’. The contacts with COVID-19-like symptoms persons were mainly with family members and friends (54.9%), co-workers (25.2%), and classmates for students (9.0%) as per stated quotes (Table 8). Burial places were also reported to have contributed to further spread of COVID-19. Due to cultural practices in Africa where bereaved members of families and communities come together during burial functions, this later resulted into congestion without adhering to SOPs.	‘…*We have recorded quite several COVID-19 cases from our markets …I’m talking about vendors, not even the customers but the vendors…’* DHO, District W. *‘You know when the STI Secretariat allowed food markets to operate but was not strict on this issue of attendance in the market. The vendors still moved to their homes and interacted with people in the market then they go and stay with their families….’* LC5, District S. *‘…And then also bars, you find that most of them are still stealthily operational and those are areas that increase spread faster.’* LC5, District S. *‘…Last time they said that bars are closed but they are very open, a drunkard can’t put on a mask. So, all these things lead to an increase in cases. One person can infect. 100 people when they are together….’* IDI Female, District M. *‘…They tell them …not to go for burials, and they don’t listen because they want to go and bid farewell to their community member and you just wonder because the person has died of COVID-19. And we know that at the burial of a COVID victim, the chances of having other infected persons are high hence spreading infection in the whole community. There was a burial around here and people slept over saying that it’s impossible not to do it.* *Even if you advise them to let a few stay, they don’t listen and you wonder if they are all going to be tested or not and you know at least 10 are infected. Because they cuddled the widow, welcomed her with hugs, and they sat in house….’* VHT, District A.
Myths, Misinformation ‘Influence of negative social media messaging’ Respondents of the KIIs reported that some members of the communities studied believed there was no COVID-19 and therefore ignored observation of SOPs while others depended on the fake social media news to inform their response to instituted SOPs	*‘…the community in the district still say there is no COVID…and they do not put on masks….’* IDI Male, District B. *‘…Another driver is that information and technology that has given freedom to people to publish anything on COVID-19 yet social media tends to be highly consumed by the community….’* District Surveillance Focal Person, District T.
Politics ‘Political Campaigns’ Respondents reported that the political season that started in the country was undergoing the first wave of COVID-19 and could have facilitated transmissions triggering the second wave of COVID-19.	*‘…you see, the campaigns were the key drivers of the second wave….’* District Medical Officer, District P.
Schools ‘Increasing COVID-19 positive cases’ Respondents mentioned that the number of COVID-19 cases among the current school pupils and students had increased and were being under-reported. Upon closure of the schools as part of the lockdown instituted early in June 2021, some pupils and students from boarding schools who were asymptomatic for COVID-19 returned home and unknowingly infected members of their families.	*‘… The number of cases that came from school are increasing because in terms of positivity the rate was at around 70% in that out of every ten individuals we were testing from the schools, seven were positive.’* DHO, District W.
Weak Health Systems ‘Inadequate funding of COVID-19 control measures’ Respondents reported inadequate resources in most health facilities across the country led to most of the confirmed COVID-19 cases being sent home for HBC management. This led to having so many positive COVID-19 cases in the communities that might have led to rapid spread of infections in the community, highlighted by high positivity rates recorded in June 2021 (Table 8). Furthermore, respondents castigated that lack of resources for nationwide sensitisation, asymptomatic infected health workers returning to their families, congestion in lower health facilities, limited testing centres, and congestion at various trade points exchanging money and goods were among the drivers of COVID-19 in the second wave.	*‘…the factor of inadequate resources to confine positive cases became key in spreading the infection….’* Resident District Commissioner, District H. *‘…inadequate resources for sensitization because the rural populace took it as a disease for the urban. ‘That’s your disease.’ And indeed, if you go to the rural areas, there’s totally no SOPs observed. So inadequate sensitization in that regard.’* District Health Officer, District S. *‘…we have seen health workers themselves getting infected. Aaaah. maybe they are not protected, health workers some of them don’t have PPEs, they don’t have what to use, they don’t have gloves, uhmm, and yet they really see patients. For that reason, we have seen health workers who have tested positive. Probably they are also the agents of spreading the disease.’* District Laboratory Focal Person, District A. *‘…On the medical perspective, it is lack of machines to use, the PPEs and the rest. That has been one of the factors. You find us working 3 to 4 days but without a mask.…’* Laboratory Focal Person, District M. *‘…….just before we got the kits yesterday, people we saw* *had all the signs of COVID, they wanted to test but they could not test, so aanha limited availability of testing points could also have been a driver because some people have signs but for as long as they have not tested positive they will not isolate. They put others at risk and yet they know their status….’* Assistant District Health Officer, District A. ‘…*Then also in our health centers there is a lot of congestion. These are areas that increase spread faster. Most health centers IIs and IIIs offer free health services so whoever believes has a challenge goes there…they tend to handle other cases such as malaria, they don’t handle COVID. Regardless a patient can spend the whole day with people at the facility hence spreading the disease….’* LC5, District S.
Money (Exchange of goods) ‘Inadequate hand and mask hygiene’	*‘…the main cause might be money since exchange of money from one person’s hand to another happens when we are buying and/or selling stuff leading to infection.* *Also, there are some people, including us who keep their masks in the same bag where money is kept, money one has just received from somebody they do not know….’* IDI Male, District W.

## Discussion

In this study, we assessed the factors associated with the observed wide spread and impact of COVID-19 among the Ugandan population during the second wave of SARS-CoV-2 infections between March and June 2021 from 10 districts in Uganda. In the second wave of COVID-19, we had a slightly higher proportion of female cases compared to males. Our results represent a shift from the first wave, where males were the most affected [22, 23] as has been reported elsewhere [24, 25].

We found that the majority of the cases reported having several and varying symptoms during the course of the disease where most of them reported cough, headache, runny nose, fever, and general body weakness as previously reported [26, 27]. We further observed poor healthcare-seeking behaviours among the COVID-19 cases in our study, where 18.3% of cases never sought care at all and 81.7% sought care after experiencing COVID-19 symptoms. Even those who sought healthcare went after experiencing advanced stages of the disease with severe symptoms like difficult breathing as verified by the information from in-depth interviews. Whereas the studied COVID-19 cases presented themselves for testing having experienced COVID-19-like symptoms, the biggest proportion (91.3%) were sent back home for home-based care management (HBCM) as designated COVID-19 treatment units were overwhelmed with severe cases. The Ministry of Health had established and approved HBCM guidelines [28, 29] and rolled them out to decongest designated COVID-19 treatment units. Unfortunately, the HBC guidelines were rolled out without a proper strategy for implementation and supervision, and hence families with COVID-19 cases were not sure of what to do, lacked supervisory support, and were not able to adhere to SOPs within the guidelines. Our findings are in agreement with other studies conducted in the United Kingdom that showed that women were twice more likely to get COVID-19 [30], although it differs from another study in China where it was found that most of the affected persons were aged 50–55 years old [31]. Such discrepancies in studies could be explained by the fact that there is previously documented high care-seeking behaviour exhibited by women than men [32, 33]. Potentially we could see otherwise a different impression if all gender sought care the same way, and therefore these results could be skewed and biased, and not representative of the real-life experience and distribution of COVID-19 in populations [8, 9]. There is a need for gender-specific massive sensitisation of the public about new policies on COVID-19 diagnosis, treatment, and vaccination by the relevant authorities to increase compliance and uptake of COVID-19 control measures, including the current vaccination programme and booster doses. In our study, the change in gender infection status with more females being infected and together with their social roles in families and communities facilitates close interactions with households and communities with more likelihood of increasing transmissions. We further noted increased cases among all age groups with more cases recorded in the young people aged 19 to 39 years that constituted the highest percentage (62%) of infections in the second wave. Again, our results reflect a change in the risk groups in the second wave, where young people including school-going age children were infected and probably escalated the spread of infections in their communities. Previously in several studies, the virus was more reported in adults aged 40 years and above including disease severity presentation [34]. In our current study, we found that the virus was affecting all age groups, especially the young ones. We also report mortalities ranging from 1–3.7% among the infected young ones aged 13 to 39 years, which was not the case in the first wave. We strikingly noted high cases of underlying conditions (high blood pressure and diabetes) among the young COVID-19-positive cases aged 20–39 years. This observation is surprising and may explain the increased numbers of severe cases and hospitalisations observed and reported in the second wave. Whereas it has been severally reported that COVID-19 remains limited in young ones in terms of numbers, disease presentation, and clinical outcomes, our study suggests otherwise. We think that there has been limited attention and focus on this age group as most cases would probably remain asymptomatic and rarely tested. During KIIs, it was reported that the COVID-19 positivity rate was high, up to 70%, among students returning from boarding schools upon closure of schools in the second lockdown in June 2021. Hence, our results call for a shift in outbreak response strategies to address the current disparities and prioritise women and young generations for interventions like vaccinations and specific awareness messages targeting this category to prevent further spread of infections.

Our study further noted that there is a need to have infection prevention and control (IPC) measures to mitigate health facility–acquired nosocomial infections which may arise due to less observance of IPC guidelines. In our qualitative results, the respondents expressed fear that unprotected health workers may pose a risk for COVID-19 transmission to patients during health-seeking care. The exposed health workers before testing positive continued to interact with other patients, members of their families, and communities, an exposure factor for virus transmission. Our results are in agreement with other studies, including one of the national surveys in Italy where it was found that over 74% of the people were health workers and many of them were women [35]. In China, health workers were found to be positive for COVID-19 and many of them had signs and symptoms [36]. One more critical area of concern identified during our study were social gatherings that continued to take place unabated despite government directives on social gatherings like burials, weddings, churches, bars and restaurants, salons, markets, public transport, and schools. SOPs like wearing facemasks, social distancing of at least 2 meters, minimum numbers recommended of some social functions, and hand washing with soap/sanitisers were not being observed, ignored, or even completely forgotten. Respondents of KIIs and in-depth interviews castigated that the non-adherence to SOPs for social gatherings accelerated the number of cases in most communities observed in the second wave. Even the schools that were opened in a staggered manner with prior preparations and clear instructions to curtail transmissions became a seedbed for COVID-19 transmissions. The schools flaunted instructions and some concealed information about COVID-19 cases for fear of being closed. By the time the schools were closed again in June 2021, the cases both identified and unidentified were very high and further contributed to community transmissions upon returning home. As much as our school situation and operational settings may be different with so many boarding schools compared to other regions of the world, schools (students and teachers) had been reported as one of the super-spreaders of SARS-CoV-2 [37, 38]. The social gatherings were further fuelled by stigma, social media misinformation, and falsifications that circulated widely about COVID-19 that affected many of the instituted prevention measures as also reported elsewhere [39]. At the time of the study and during the study period, COVID-19 vaccine access was extremely very low and only 4% of the studied COVID-19 cases had received two doses of AstraZeneca vaccine. At the national level, only less than 2% of the targeted population had received two doses of the vaccine [40]. Hence, the biggest percentage of the population remained naïve and vulnerable to SARS-CoV-2 infections and associated severe disease outcomes, especially among the elderly and those with comorbidities. In addition to having a vulnerable population, Uganda also registered and reported the existence of COVID-19 variants (Delta, Eta, Alpha, Beta, and local strain) in June 2021 [41, 42]. Low vaccination coverage together with the emergence of COVID-19 variants could have contributed to the high numbers of COVID-19 cases and associated mortalities registered in June 2021 alongside other factors already described in this study. Our study differed from other studies including the one conducted in the United States which showed that COVID-19 vaccination was up to 57%, with the majority of them at least receiving a single dose of vaccination during the same period of this study [43, 44].

Our study had a number of strengths. First, we visited different districts in Uganda which are geographically spaced and this gave a better picture of what was happening across the entire country. Secondly, we used both quantitative and qualitative methods, and this helped us to probe further on some salient issues that could have emerged from the quantitative findings. We also used a standard MOH case investigation tool, and this helps our results to be generalised across the country. We further visited quite a reasonable sample size that is representative of the COVID-19 cases at that time. We also conducted both bivariate and multivariate logistic regression for our project. We also had a good response rate of 93%, and this was a deliberate effort by the research team. Lastly, we interviewed frontline health workers and supervisors that helped us to get real-life facts on the spread of the disease during this period. We also acknowledge several limitations given its retrospective nature. First, we reviewed secondary information at the testing centres and laboratories, and this exposed us to incomplete and inaccurate documentation in such places. We also did not statistically determine the sample size for our study given the emergency we were in. Second, we had few confirmed cases by post-mortem as this was done for a few financially stable persons. We also failed to document the age and gender of the 7% non-respondents during our data collection, and yet this would inform the presentation of results. Lastly, we interviewed people during the key informants and in-depth interviews after they had gone through COVID-19 signs and symptoms, and this is likely to have contributed to recall bias among the participants.

## Conclusions

Our research found that various factors, including demographic, patient, health facility and service, social, and economic-related elements, contributed to the emergence and persistence of the second wave of COVID-19 from March to June 2021.

Specifically, young, asymptomatic individuals not under home-based care, those working or studying in schools, and those who were not vaccinated were major drivers of the second wave. To effectively manage future waves of COVID-19, proactive efforts should be made to enhance home-based care services, strictly observe SOPs in schools, and increase vaccination rates. To continue protecting communities from emerging variants of SARS-CoV-2, all stakeholders, including policy makers, healthcare workers, non-governmental organisations, the public, and researchers, must work together to implement vigilant surveillance services at the community and home levels and increase vaccination uptake. This will help to minimise the health, social, and economic impacts of COVID-19.

## Data Availability

The data collected using different forms and associated data for results presented in this manuscript can be downloaded at the mWater Portal on request.

## Appendix B: Composition of Key Informant and Focus Group Discussion Team members

**Table:**

S/N	Key informants	In-depth Interviewees
01	District Chairpersons	Community members
02	Resident District Commissioners	Community health workers/Village Health Team
03	District Health Officers	School headteachers or representatives
04	District Surveillance Focal Persons	Religious leaders
05	District Laboratory Focal Persons	Cultural leaders or their representatives
06	Leaders of different health facilities	Local Council Chairpersons
07	COVID-19 leaders	
08	Members of district epidemic taskforces	

## Appendix C: Key informant, in-depth interview guide, and consent form key informant guide (sub-study 2)

For this objective, key informant interviews will be conducted among the District Task Force members: RDC, L.C V, DHO, DSFP, DLFP; Head of case management (private and public): VHT, L.C.1.

What in your opinion is the response to COVID? (probe about the tenets of the response)

In your opinion, how would you generally describe and rate the response to COVID-19 in your district?

Have you played any role in the response? (probe on their experience working in the response, what has your role been, challenges, mitigation strategies, support network)

Do you have any comment on the COVID-19-related deaths? (probe on community and facility deaths)

Describe the situation of COVID-19 vaccination in your community (vaccine hesitancy, uptake, availability, myths, side effects…).

In your opinion, what are the drivers of the COVID-19 infections (risk factors/ factors associated with infections)

What are the treatment places/centres for COVID-19 in your district/ community? (probe: describe their location, capacity for case management).

What has worked well in the response? (probe on availability of equipment, vaccines, treatment beds, ICU capacity, logistics, health workers, laboratory capacity)

What has not worked well? What can be done to improve the current and future responses?

## Appendix D: In-depth Interview Guide

We shall conduct four in-depth interviews with participants that have ever contracted COVID-19; two men and two women. For either gender, we shall have two participants that will have been treated in a health facility and those that underwent home-based care.

What in your opinion is the response to COVID? (probe about the tenets of the response)

In your opinion, how would you generally describe the response to COVID-19 in your district?

Describe your experience when you contracted COVID-19 (when did you contract COVID-19, where do you think you contracted it from, did you test, for how long were sick, where were you treated from)

Do you have any comment on the COVID-19-related deaths (probe on community deaths)

Describe the situation of COVID-19 vaccination in your community (vaccine hesitancy, uptake, availability, myths, side effects…).

In your opinion, what are the drivers of the COVID-19 infections in your community?

What are the treatment places/centres for COVID-19 in your district/community? (probe: describe theirs)

What has worked well in the response? (probe on availability of equipment, vaccines, treatment beds, ICU capacity, logistics)

What has not worked well?

What can be done to improve the current and future responses?

## Appendix E: Consent form Outbreak Investigation and assessment of the challenges faced by the Public Health system in containing SARS-CoV-2 in Uganda, June 2021

### Introduction

My name is………………………………………, a member of a team conducting a COVID-19 outbreak investigation and an assessment of the challenges faced by the Public Health system in containing COVID-19 in Uganda, June 2021 under the Presidential Scientific Initiative on Epidemics (PRESIDE) under the Science, Technology and Innovation Secretariat (www.sti.go.ug). You are selected to participate in this investigation to share your experience with COVID-19 infection. This study will seek your views about the drivers, strengths, gaps/challenges faced by the Public Health system in containing COVID-19 in Uganda.

Your views will enable the COVID-19 National Response team understand why the country is experiencing a rapid increase in the transmission of this disease so as appropriate actions/interventions can be put in place to curb the infections.

### Confidentiality

The information you will share with investigation team shall be kept very confidential. All your responses will be kept anonymous and your personal identifiers like names, telephone, gender, position/role/designation will not appear anywhere in the final report. For the key informant and in-depth interviews, we request to record your audio submission(s) to enable later transcribing which will enable report generation. Regarding potential benefits, there will be no monetary benefits for you to participate in this study, however, your shared experience will contribute to the National Task Forces’ efforts in developing and implementing interventions to respond to the COVID19 Pandemic.

### Potential Risks and Discomfort

You have a right to answer or decline responding to some questions which you may not be comfortable with. You may choose to end the interview or withdraw from this investigation at any during its course.

Do you have any questions?

Do you agree to participate in this study?

If yes,

Signature_______________________________.

### Contact Person for Questions

If you have further questions or inquiries about this investigation, feel free to contact you may contact the following lead researchers on this study: Abel Wilson Walekhwa, +256752206865.
